# In-Plant Protection against *Helicoverpa armigera* by Production of Long hpRNA in Chloroplasts

**DOI:** 10.3389/fpls.2016.01453

**Published:** 2016-09-29

**Authors:** Julia Bally, Glen J. McIntyre, Rachel L. Doran, Karen Lee, Alicia Perez, Hyungtaek Jung, Fatima Naim, Ignacio M. Larrinua, Kenneth E. Narva, Peter M. Waterhouse

**Affiliations:** ^1^Centre for Tropical Crops and Biocommodities, Queensland University of Technology, BrisbaneQLD, Australia; ^2^School of Molecular Bioscience, University of Sydney, SydneyNSW, Australia; ^3^Dow AgroSciences, IndianapolisIN, USA

**Keywords:** *trans*-kingdom RNAi, chloroplast transformation, hpRNA, insect control, acetylcholinesterase, *Helicoverpa armigera*

## Abstract

Expressing double-stranded RNA (dsRNA) in transgenic plants to silence essential genes within herbivorous pests is referred to as *trans*-kingdom RNA interference (TK-RNAi) and has emerged as a promising strategy for crop protection. However, the dicing of dsRNA into siRNAs by the plant’s intrinsic RNAi machinery may reduce this pesticidal activity. Therefore, genetic constructs, encoding ∼200 nt duplex-stemmed-hairpin (hp) RNAs, targeting the acetylcholinesterase gene of the cotton bollworm, *Helicoverpa armigera*, were integrated into either the nuclear or the chloroplast genome of *Nicotiana benthamiana.* Undiced, full-length hpRNAs accumulated in transplastomic lines of *N. benthamiana* and conferred strong protection against *H. armigera* herbivory while the hpRNAs of nuclear-transformed plants were processed into siRNAs and gave more modest anti-feeding activity. This suggests that there is little or no RNAi machinery or activity in the chloroplast, that hpRNAs produced within this organelle do not enter the cytoplasm, and that oral delivery of chloroplast-packaged intact hpRNA is a more effective means of delivering TK-RNAi than using nuclear encoded hpRNAs. This contrasts with a recently reported correlation between siRNA expression and effectiveness of TK-RNAi targeting the chitinase gene of *H. armigera*, but is consistent with reports of efficient TK-RNAi by dsRNA generated in chloroplasts by converging promoters flanking a pest gene sequence and from very small (21 nt-stem) hpRNAs resembling artificial miRNAs. Here we demonstrate that hpRNAs, constructed along the conventional design principles of plant RNAi constructs but integrated into the chloroplast genome, are stable and effective over multiple generations, and hold the promise of providing durable pest resistance in crops.

## Introduction

The RNA interference (RNAi) pathway occurs in plants and animals and has been exploited to specifically down-regulate gene expression in a wide range of research and applications (Wesley et al., 2001; Eamens et al., 2008; Meyer, 2008; Zhu et al., 2014). It also facilitates *trans*-kingdom RNAi (TK-RNAi), commonly referred to as environmental or host-delivered RNAi (eRNAi; HD-RNAi), whereby hpRNA, dsRNA, or small RNA synthesized in one organism silences the target gene in another. TK-RNAi was first observed when feeding *Caenorhabditis elegans* on *Escherichia coli*, expressing *unc21*-sequence dsRNA, induced a twitching phenotype (Fire et al., 1998). The principle of TK-RNAi has been replicated many times since in the search for sustainable pest resistance in plants (Miller et al., 2012).

Most TK-RNAi for agricultural pests has relied on nuclear expression of long hairpin (hp) RNA, with its transport into the plant’s cytoplasm, and endogenous RNAi machinery processing it into ∼21 nt siRNA, before ingestion by the target pest. This approach has given strong growth inhibition and lethal phenotypes in the Coleoptera, e.g., western corn rootworm, *Diabrotica virgifera* (Baum et al., 2007; Bolognesi et al., 2012), and Colorado potato beetle (CPB), *Leptinotarsa decemlineata* (Zhu et al., 2011). However, TK-RNAi against Lepidoptera such as *Helicoverpa armigera* has given mixed results (Price and Gatehouse, 2008; Terenius et al., 2011; Burand and Hunter, 2013; Lim et al., 2016). One suggestion has been that this variation in efficacy is due to different levels of unprocessed dsRNA or hpRNA being available for ingestion by the pest and that this depends on the transcription rate of the hpRNA in relation to the processing rate of the cell’s RNAi machinery (Gordon and Waterhouse, 2007). Indeed, producing unprocessed hpRNAs in dicer-mutant plants has given stronger *trans*-kingdom RNAi than in wild type plants (Mao et al., 2007). The use of RNAi-deficiency in plants may increase the effectiveness *trans*-kingdom RNAi pest protection, but it is an unlikely scenario for field applications because such plants would be hyper-susceptible to viral pathogens and developmentally defective due to compromised micro- and tasi-RNA activity. Therefore, we sought to test whether expressing hpRNAs in the plant’s chloroplasts would provide insect protection; this cellular compartment may have little or no RNAi-like activity and therefore not have the collateral damage of the defective RNAi approach.

Two similar methodologies have recently been reported, but one used dsRNA expressed from two convergent promoters (Zhang et al., 2015) and the other study used very short (∼21 nt-stem) hpRNAs which appear to undergo processing (Jin et al., 2015). Our original hpRNAi construct designs (Smith et al., 2000; Wesley et al., 2001), have become the standard for plant gene silencing, use hpRNAs with “arms” of 120–1000 nt flanking an intron “loop.” Therefore, we sought to test whether transforming the chloroplasts of *Nicotiana benthamiana* plants with similarly designed (∼200 nt stem) hpRNA constructs, targeting the acetylcholinesterase (ACE) gene of *H. armigera*, would be stable and confer insect protection. We compared the processing and efficacy of these hpRNAs produced in the nucleus or the chloroplasts and, in order to make this comparison, we also examined whether the developmental age of the transgenic plants could mask or enhance the effects of TK-RNAi.

## Materials and Methods

### Insect Feeding Bioassay

*Helicoverpa armigera* larvae were raised from a culture maintained by ABA Biologicals, Glenvale, QLD, Australia. The eggs were placed on artificial diet and allowed to hatch in a growth cabinet set at 26°C and 60% humidity. Assays were performed with newly hatched larvae (<10 h old). Each larva was transferred using a moistened soft paintbrush to the appropriate petri dishes containing a seedling (1 and 2 weeks old plants) or a single leaf (3, 5, and 7 weeks old plants) cut from plants grown at 23°C under a 16-h photo-period and an 8-h dark period in an environmentally controlled glasshouse. For each assay, 5 or 10 larvae were placed in the petri dishes and the experiment was monitored by assessing leaf damage and larva growth weight after 4 days.

The plants used in this study were corn *Zea mays*, cotton *Gossypium hirsutum*, and six isolates of *N. benthamiana*: the laboratory isolate a wax-less, trichome less variety and five wild-type isolates with thicker leaf shape and dense trichomes organization (Bally et al., 2015)

### Generation and Molecular Characterization of Transplastomic Plants

The 189 nt stem sequences of the ACE gene of *H. armigera* (Ace; NCBI accession AF369793) was amplified by PCR using *H. armigera* cDNA, and the primers pACE189f/pACE189r. The primers used in this study are listed in Supplementary Table S2.

The hpRNA expression sequences were made with the amplified ACE sequences, self-complementary 189 nt. sense (SE)/antisense (AS) stem configuration with the stems separated by an intron-based spacer of ∼1599 nt., using the ‘Golden Gate’ (GG) assembly method (Yan et al., 2012). The resulting product was inserted behind the CaMV 35 s promoter in a binary vector (Coutu et al., 2007) to create the nuclear dsRNA expression control vector, hpACE-n and behind the Prrn in the chloroplast transformation vector pPRV312L (NCBI accession DQ489715.1) (Chakrabarti et al., 2006). Plastid transformation and plant selection were carried out essentially as described by Svab and Maliga (1993). Briefly, sterile *N. benthamiana* plants were grown in solid MS media supplemented with 30 g/L sucrose. The transformation was carried out by bombarding 4–5-week-old leaves (length, 4–6 cm) with gold particles coated with the appropriate vector, using a particle influx generator gun. Following incubation at 24°C in MS medium supplemented with hormones, 6-benzylaminopurine (2 mg/L) and 1-naphthalene acetic acid (0.05 mg/L) for 2 days, bombarded leaves were cut into small pieces (∼1 cm^2^) and subjected to selection on media containing 500 mg/L of spectinomycin. Resistant shoots obtained after about 6 weeks were transferred to rooting media and subsequently transplanted into soil in the glasshouse.

### DNA Purification and RNA Purification

Larvae and plant tissues were ground to a fine powder under liquid nitrogen. Total DNA was extracted from the young leaves of soil-grown plants using the CTAB method (Clarke, 2009). Total RNA was extracted from 0.2 mg of sample using TRIzol^®^ reagent (Thermo Fisher Scientific Inc., Waltham, MA, USA), and purified using the RNeasy Plant Mini Kit (QIAGEN) following the protocols supplied by the manufacturers. Total DNA and total RNA quality was assessed with a Nanodrop 2000 UV-VIS Spectrophotometer (Thermo Fisher Scientific, Inc., Waltham, MA, USA).

### RT-PCR/qPCR

Acetylcholinesterase transcript levels in the different samples were analyzed by quantitative real-time real-time reverse-transcription PCR (RT-PCR) with a PTC-200 Thermal Cycler (MJ Research/Bio-Rad). In brief, synthesis of cDNA for RT-PCR was performed using SuperScript^®^ III Reverse Transcriptase (Life Technologies) following the manufacturer’s instructions. RT-PCR was performed using Brilliant III SYBR^®^ MM according to the Agilent Technologies protocol.

### Southern Blot Analyses

Five micrograms of total DNA was digested with SmaI, separated on a 8% agarose gel and transferred to a Hybond N+ (Amersham) membrane and cross-linked to it by UV treatment. The probe used for Southern blot analyses was amplified using the primer pair 5′- ATGGACGGTAGTTGGAGTCG -3′ and 5′- TCCTTGGGGTGATCTCGTAG -3′ and the resulting PCR fragment purified and radiolabeled with ^32^P by random priming using the MEGAPRIME Kit (Qiagen). The membrane was hybridized overnight at 65°C with the labeled riboprobe and exposed to FujiFilm X-100 for 1 h.

### Northern Hybridization

#### Small RNAs Detection

A 17 % PAGE gel was loaded with approximately 20 ug of total RNA per lane and run at 200V for 4 h. After staining with ethidium bromide to confirm the relative loadings and appropriate size separation, the RNA was electro-transferred at 45 V for 1 h onto Hybond N+ (Amersham) membrane and cross-linked to it by UV treatment. The membrane was incubated at 42°C with a ^32^P-labeled probe transcribed from the hp arm sequence and exposed to FujiFilm X-100 for 16 h.

#### High MW RNAs Detection

Approximately 20 ug of total RNA was separated on a 1.4 % agarose formaldehyde gel. After staining with ethidium bromide to confirm the relative loadings and appropriate size separation, the RNA was transferred onto Hybond N+ membrane (Amersham) by capillary transfer blotting with 1X MOPS and cross-linked to it by UV treatment. The membrane was hybridized overnight at 68°C with a ^32^P -labeled riboprobe transcribed from the hp arm sequence and exposed to FujiFilm X-100 for 1 h.

### Acetylcholinesterase Enzymatic Assay

After 4 days of feeding experiment, *H. armigera* larvae were homogenized in 100 μl of ice-cold extraction buffer (100 mM Tris–HCl pH 8.0, 200 mM NaCl, and one tablet per 10 mL or protease inhibitor – Roche) then 15 min centrifugation at 13,000 rpm at 4°C. The supernatant was recovered, measured by Bradford assay (Bradford, 1976), and adjusted to 0.5 mg protein/ml by the addition of extraction buffer. The protease activity in the extracts was then determined using an ACE Assay kit (Abcam – ab138871).

## Results

### *Helicoverpa armigera* Feeding Bioassay

**FIGURE 1:**
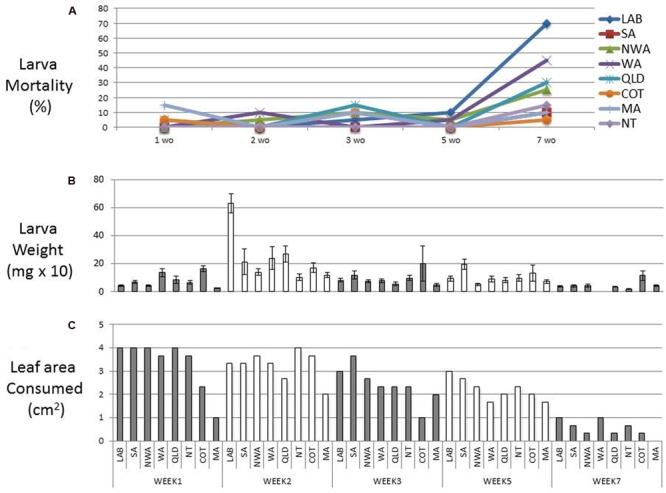
**Feeding bioassay.** Mortality **(A)** and average weight **(B)** of first instar *Helicoverpa armigera* after 4 days of feeding on 1, 2, 3, 5, and 7 weeks old plants. All data are means ± SD (*n* = 30). The leaf area consumed for each feeding treatment is represented in **(C)**. Six different accessions of *Nicotiana benthamiana* [one LAB strain and five wild strains (SA, NWA, WA, QLD, NT; (Bally et al., 2015), cotton (COT) and maize (MA), were also tested].

The age, tissue, and plant species used in *trans*-kingdom RNAi bioassays has varied greatly. Moreover, preliminary experiments on our favored model plant, *N. benthamiana*, suggested that leaves from 6-week-old plants were unpalatable or toxic to *H. armigera*. To define better bioassay conditions, we evaluated *H. armigera* survival and weight-gain on leaves from plants of different ages. Six different accessions of *N. benthamiana* (one LAB strain and five wild strains; Bally et al., 2015), as well as cotton and maize, were tested. Samples of leaf tissue from plants 3, 5, and 7 weeks post germination, and whole 1 and 2 week-old seedlings were placed into Petri dishes with 30 *H. armigera* neonates. After 4 days on the tissues from 1, 2, 3, or 5 week-old (wo) *N. benthamiana* plants, about 90% of the larvae still survived (**Figure 1A**). However, the consumption of leaves from 7 wo plants was minimal, the larval growth-rate was impaired, and their survival rate dropped to about 50% (**Figures 1A–C**). Larvae feeding on two wo *N. benthamiana* LAB seedlings had the most obvious weight-gain, and this trend was also observed for almost all of the *N. benthamiana* ecotypes and the other species. Therefore, transgenic and transplastomic plants were made using the LAB strain, almost all bioassays were done with 2 wo seedlings, and none utilized leaves from plants over 5 wo.

### Design of hpRNA against Acetylcholinesterase in Chloroplasts

**FIGURE 2:**
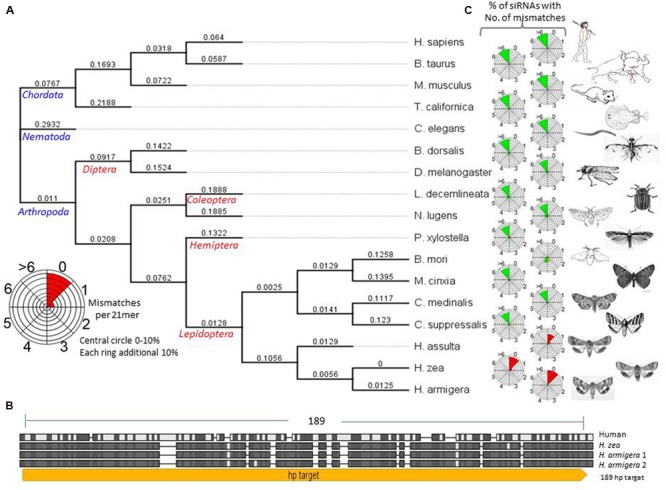
**(A)** Acetylcholinesterase (ACE) phylogenetic tree based on nucleic acid sequences made with the neighbor-joining method. *Bombyx mori* (*B. mori*; DQ115792), *Bos taurus* (*B. Taurus*; NM_001076220), *Caenorhabditis elegans* (*C. elegans*; X75331), *Drosophila melanogaster* (*D. melanogaster*; X05893), *Helicoverpa armigera (H. armiger;* AF369793*), Helicoverpa assulta* (*H. assulta*; AY817736), *Helicoverpa zea* (Supplementary Figure S1), *Homo sapiens* (*H. sapiens*; M55040), *Leptinotarsa decemlineata* (*L. decemlineata*; L41180), *Mus musculus* (*M. musculus*; X56518), *Nilaparvata lugens* (*N. Lugens*; AJ852420), *Torpedo californica* (*T. californica*; X03439*), Chilo suppressalis (C. supressalis*; EF470245.1), *Cnaphalocrocis medinalis (C. medinalis*; FN538987.1), *Melitaea cinxia (M. cinxia*; GQ489250.1), *Plutella xylostella (P. xylostella*; AY032625.1). **(B)** *H. armigera* ACE nucleotide sequence showing the hairpin target site, alignment of ACE1 nucleotide sequences from *H. sapiens, H. zea, H. armigera* and ACE2 of *H. armigera*. Conserved residues are enclosed in black/gray boxes, hairpin target region indicated in yellow (hp189). See Supplementary Figure S1 for details of sequence. **(C)** Radial siRNA relationship icons for each species calculated by TK-siRNA software at benthgenome.com. They indicate the percentage of possible siRNAs from the *H. armigera* ACE hpRNA sequence that have 0, 1, 2, 3, 4, 5, 6, or >6 mismatches with the corresponding gene in the different species. Twenty-one nucleotides of siRNA sequences with 0, 1, or 2 mismatches are expected to target cleavage of the target gene (and shown in red), those with >3 mismatches are shown in green and expected to have no significant RNAi effect. Those shown in orange are placed in the category of possibly having an RNAi effect. Each successive circle represents a 10% bin from 0–10 to 90–100%. For example: a completely red sector for 0 mismatches (e.g., for *H. armigera*) represents 100% of the potential siRNAs are predicted to direct an RNAi effect and a completely green sector for >6 mismatches (e.g., for *H. sapiens*) represents 100% of the potential siRNAs are predicted to not direct an RNAi effect. The prediction about degrees of mismatch having an RNAi effect comes from the results shown in Supplementary Figure S2.

**FIGURE 3:**
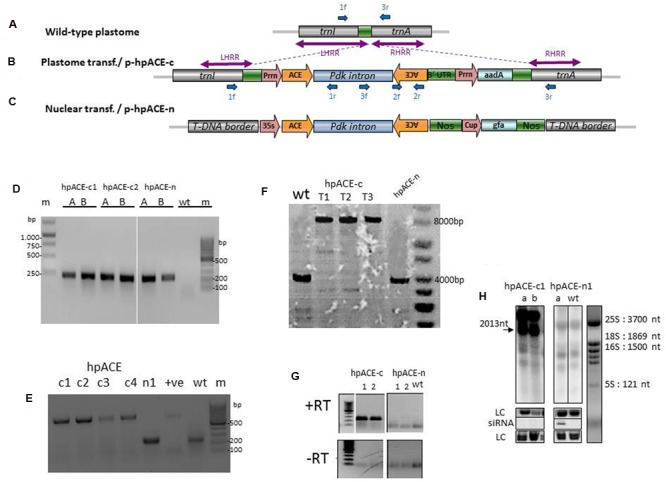
**Acetylcholinesterase hairpin dsRNA construct and evaluation of transgene integration in the plastid genome.** Targeted plastid genome region **(A)** and recombinant tobacco plastome **(B)** after recombination with transforming vector p-hpACE-c and transgene integration. *aadA*, spectinomycin resistance gene; *Prrn*, tobacco 16SrDNA promoter; *PsbA*, tobacco psbA promoter; *rbcL*, Rubisco large subunit. LHRR and RHRR are the left and right plastid recombination regions present in the transforming vector. **(C)** Nuclear transformation vector. Forward (f) and reverse (r) primers used for PCR and RT-PCR analysis are indicated by blue arrows. **(D)** PCR analysis of transformed lines showing hairpin arm amplification using the primer pair 2f/2f and the integration location **(E)** using primer pairs 1f/1r or 1f/3r. **(F)** Southern blot showing the homoplasmy for the first, second, and third generation of transformed lines. **(G)** Reverse transcription (RT) assays and Northern blot **(H)** of RNA samples extracted from hpACE-c and hpACE-n transformed and untransformed *N. benthamiana* plants. LC; loading control.

Acetylcholinesterase is an essential enzyme for the central nervous system of *Helicoverpa armigera* and the target of many organophosphorus and carbamate insecticides (Kumar et al., 2009). Therefore, we selected the ACE gene family for *trans*-kingdom RNAi. Some domains of the ACE enzyme are conserved from insects to mammals, at the amino acid level, but regions of their mRNA sequences are highly variable (**Figure 2**; Supplementary Figure S1). The nucleotide sequences of ACE genes for *H. armigera, H. zea*, and *H. sapiens* were aligned and a sequence (189 nt) of *H. armigera* ACE2 (AF369793) with high similarity to ACE1 (AY142325) of *H. armigera* and *H. zea*, and high divergence from the human sequence (**Figure 2B**; Supplementary Figure S1) was selected for further analysis. Bioinformatic examination of this sequence using the RNAi target tester^1^ breaking the sequence down into every possible 21 nt oligomer and comparing them with the nucleotide sequences of ACE from species ranging from *H. armigera*, to humans, revealed that none of these potential siRNAs would have less than six mismatches with human genes, but more than 95% of them would recognize the *Helicoverpa* ACE1 and ACE2 genes with 0 or 1 mismatches (**Figures 2A,B**; Supplementary Figure S1). A similar analysis of synthesized dsRNA fed to the corn rootworm (Bachman et al., 2013), targeting the *snf7* gene, indicated that sequences with more than three mismatches per 21mer are unlikely to be effective (Supplementary Figure S2). From this benchmark, the selected 189 ACE dsRNA should have little or no silencing effect on either non-lepidopterans or lepidopterans other than *Helicoverpa* spp. (**Figure 2C**). Therefore, the 189 nt ACE2 sequence was used in the construction of hpRNA vectors for chloroplast and nuclear transformation of *N. benthamiana* (**Figures 3A,B**). The chloroplast hpRNA expression vector (hpACE-c) was based on pPRV312L (DQ489715.1) (Chakrabarti et al., 2006) with the arm sequences of the hpRNAs placed either side of a pdk intron and flanked by the prrn promoter- 5′-untranslated region and the rbcL 3′-UTR sequence. The nuclear hpRNA expression vector (hpACE-n) was based on pORE (Coutu et al., 2007) and contained a 35S promoter and NOS terminator (**Figure 3C**).

### Generation and Characterization of Chloroplast and Nuclear Transformants

Many plantlets (T0) were regenerated from leaves bombarded with p-hpACE-c and from leaf pieces inoculated with *Agrobacterium* harboring the hpACE-n. They were analyzed by polymerase chain reaction (PCR), for integration of the hpRNA cassette (**Figure 3D**). Further PCR analysis (**Figure 3E**) with a three primer cocktail revealed that many of the lines harbored both transformed (619 nt band from 1f+1r) and untransformed chloroplasts (226 nt band from primers 1f+3r) but with continued selection, and passage through two seed generations, homoplastic transformed plants were obtained as confirmed by Southern analysis (**Figure 3F**; Supplementary Figure S3). The faint background bands are non-specific, as is often seen in chloroplast blots (Dufourmantel et al., 2007; Zhang et al., 2015). Two independent T2 transgenic lines were selected for each construct. T2 nuclear transformants were identified as producing siRNAs by Northern blot (**Figure 3H**).

To test whether the pdk intron of the hpRNA is excised in the chloroplast, RNA was extracted from hpACEc, hpACEn and untransformed plants. The samples were analyzed by reverse-transcription PCR using primers 3f+2r. The hpACE-c lines gave amplicons encompassing part of the intron and the rear stem sequence (**Figure 3G**), suggesting that they are producing hpRNA transcripts that are neither diced nor spliced. This was further substantiated by Northern blot (**Figure 3H**). The full length of intron-containing hpRNAc transcript with its rbcL 3′-UTR should be 2013 nt and membranes bearing samples from hpACEc plants probed with radio-labeled sequences, spanning the 189 nt hpRNA-arm sequence, produced such a band (**Figure 3H**). A larger molecular weight band was also detected and this may be the result of transcriptional read-through, which is commonly seen in chloroplasts. In conjunction with the lack of detectable siRNAs in extracts from these plants (**Figure 3H**), the abundant presence of the full-length hpRNA indicates that chloroplast-expressed hpRNA is not processed by RNAi machinery.

Altogether, this suggests that the hpRNA produced in transplastomic chloroplasts is not subjected to RNAi-like activity in the chloroplast nor is it being transported to extra-chloroplastic places where RNAi-mediated processing could occur.

### Bioassay for Anti-Feeding Activity of hpACE and the Effect on ACE Activity in Larvae

**FIGURE 4:**
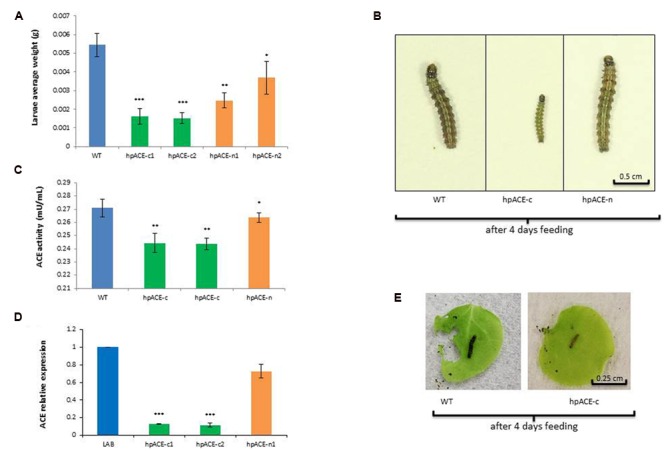
***Helicoverpa armigera* dsRNA feeding assay.** Feeding assays of *H. armigera* on transplastomic (hpACE-c) and nuclear (hpACE-n) transgenic *N. benthamiana*. Representation of *H. armigera* average weight **(A)** and size **(B)**, ACE activity **(C)** and relative expressive **(D)** after 4 days of feeding on WT, transplastomic hpACE-c and nuclear hpACE-n transformants. All data are means ± SD (*n* = 30) after 4 days of caterpillars feeding on 2 week old seedlings. Significant differences to wild type were identified by analysis of variance using ANOVA.^∗, ∗∗^, and ^∗∗∗^ indicates a significant difference at *P* < 0.5, 0.1, and 0.001, respectively. An example of feeding assay with 3 wo detached leaves is represented in **(E)**.

Neonate *H. armigera* larvae were allowed to feed on WT, hpACE-n or hpACE-c two wo seedlings for 4 days and then individually weighed (**Figure 4**). Larvae exposed to the hpACEc lines had very significantly (*p* < 0.05) reduced weight-gain over to those fed on hpACEn seedlings which, in turn, had significantly (*p* < 0.5) reduced weight-gain over those fed on wild type plants (**Figures 4A,B**; Supplementary Table S1). To test whether the anti-feeding effect of hpACE-c plants could be directly attributable to the ingested hpRNA directing down-regulation of the target ACE genes in *H. armigera*, the messenger RNA levels of the gene and the overall activity of the enzyme were measured (**Figures 4C,D**). In larvae fed on hpACEn seedlings, the levels of ACE mRNA and ACE activity were lower, but not significantly (*p* > 0.5), than those of larvae fed on WT seedlings. In contrast, the ACE mRNA levels in larvae fed on hpACE-c seedlings were reduced by ∼fivefold and the ACE enzyme activity by 30–40% (**Figure 4C**). When the bioassay was repeated with leaves of 3 wo plants, the anti-feeding activity was clearly apparent (**Figure 4E**).

## Discussion

Several biotechnological and chemical approaches are used to control crop infestations by the *H. armigera* larva but further effective strategies are needed to keep pace with ever-developing resistance (Lim et al., 2016). TK-RNAi is emerging as a promising way to defend crops against herbivorous pests but, the results have been variable (Price and Gatehouse, 2008; Burand and Hunter, 2013; Lim et al., 2016). One source of this variation includes the assay conditions and, while the effect of larval age and developmental stage has been well studied and optimized (Raubenheimer and Browne, 2000; Shelomi et al., 2010), less has been done to establish the best age and tissue of the plant for feeding bioassays. Therefore, before comparing plastid and nuclear delivered TK-RNAi, we examined the effect of the age and species of the plant material on *H. armigera* feeding. This showed that the larva’s feeding, development and weight-gain was optimal when fed on 2 week-old seedlings and greatly impaired by feeding on leaves from 7 wo plants of most isolates of *N. benthamiana* or maize. The LAB isolate of *N. benthamiana* is a widely used model plant for biotechnology applications such as metabolic engineering, vaccine production, plant–microbe interaction analyses and gene silencing (Bally et al., 2015). It has a short life cycle and both its chloroplast and nuclear genomes are easily transformable and well annotated^2^^,^^3^ therefore we used 1–5 wo *N. benthamiana* LAB seedling/leaf tissues when comparing TK-RNAi approaches.

Although there have been reports of a general correlation between higher levels of plant-produced siRNA and more effective TK-RNAi, it has been proposed that the higher levels of unprocessed long dsRNA give this effectiveness (Gordon and Waterhouse, 2007). Chloroplasts are ideal hosts for high-level expression of transgenes (Bally et al., 2009; Kim et al., 2009). Furthermore, none of the known components of RNAi machinery are encoded by the chloroplast genome or transported to this compartment. Consequently, dsRNAs purposely expressed there might accumulate to high levels and remain unprocessed. Indeed, when we transformed a hpRNA construct into the nuclear genome of *N. benthamiana*, the hpRNA was processed into siRNAs (as expected) whereas similar constructs in transplastomic plants gave high levels of unprocessed transcript and no detectable siRNAs.

Both the nuclear and chloroplast constructs were based on our widely adopted original hpRNA designs for silencing plant genes (Waterhouse et al., 1998; Smith et al., 2000; Wesley et al., 2001). They both included the pdk intron as a spacer between the hpRNA “arms,” even though it is unlikely to be spliced in the chloroplast, and targeted a ∼200 nt sequence in the *H. armigera* ACE gene. Two similar approaches for chloroplast-expressed TK-RNAi against insect larvae have recently been described, but they employed more unusual hp/dsRNA designs. Zhang et al. (2015), examined using convergent promoters to produce dsRNA and obtained good protection of transplastomic potato plants from the CPB using this strategy (Zhang et al., 2015). They also made some hpRNA constructs but did not test them for insecticidal activity. Jin et al (2015) used a design that produces 21 nt stem – 9 bp loop hairpins reminiscent of the shRNA constructs used in animal RNAi (Paddison et al., 2002); they targeted the *P450, Vac ATPase A* and *chitin synthase B* genes of *H. armigera* and in transplastomic cotton significantly affected the larvae’s development and pupation. Our hpRNA constructs appear to be stable in chloroplasts for at least three seed generations, and give strong protection against the targeted herbivorous pest. However, the maximum size of hpRNA cassette tolerated by a chloroplast genome remains to be determined. The protection was visible from the reduced size and viability of the feeding *H. armigera* larvae and the reduced damage they caused to the leaves. It is acting through inhibition of the target gene’s function, as both the mRNA level and enzyme activity of the ACE were greatly reduced in the larvae feeding on hpACE-c material compared to those fed on wt leaves or seedlings. It may also have an additional effect of increasing the larvae’s susceptibility to organophosphorous and carbamate insecticides, but this remains to be tested.

One of our nuclear-transformed hpACEn lines gave a statistically significant reduction in the growth rate of *H. armigera*. This is consistent with the findings of Mamta et al. (2016) who observed an anti-*Heliothis* effect with a similar design against its chitinase gene, but it was much less effective than its transplastomic counterpart. Whether this increased potency is due to compartmental prevention of plant dicers and argonautes processing the hpRNAs and sequestering siRNAs or whether the membranes of the chloroplast act as a protective barrier against the nucleases in the pest’s gut, allowing more intact hpRNA to reach the insect’s cells, will be interesting to investigate. It is also unclear which, if any, of the three approaches (very small hpRNAs, dsRNA produced by convergent promoters, or conventional hpRNAs) is more efficacious. What does appear to be clear is that the delivery of hpRNA or dsRNA to herbivorous pests via the chloroplast enhances the potency of lepidopteran TK-RNAi and promises to be a very useful method for plant protection.

## Author Contributions

JB, GM, KN, and PW conceived and designed the study. JB, GM, RD, KL, and AP performed the experiments. JB, GM, IL, KN, HJ, FN, RD, and PW contributed to the data analysis. JB and PW wrote the manuscript.

## Conflict of Interest Statement

The authors declare that the research was conducted in the absence of any commercial or financial relationships that could be construed as a potential conflict of interest.

## Footnotes

1 www.benthgenome.com

2 www.benthgenome.qut.edu.au

3 http://solgenomics.net
