# Supplementary material for: In-Plant Protection against Helicoverpa armigera by Production of Long hpRNA in Chloroplasts
Source: Front Plant Sci. 2016 Sep 29;7:1453. doi: 10.3389/fpls.2016.01453 (PMC5040858; doi:10.3389/fpls.2016.01453)
Supplement: Supplementary file 1 [file Presentation_1.PPTX]

## Slide 1
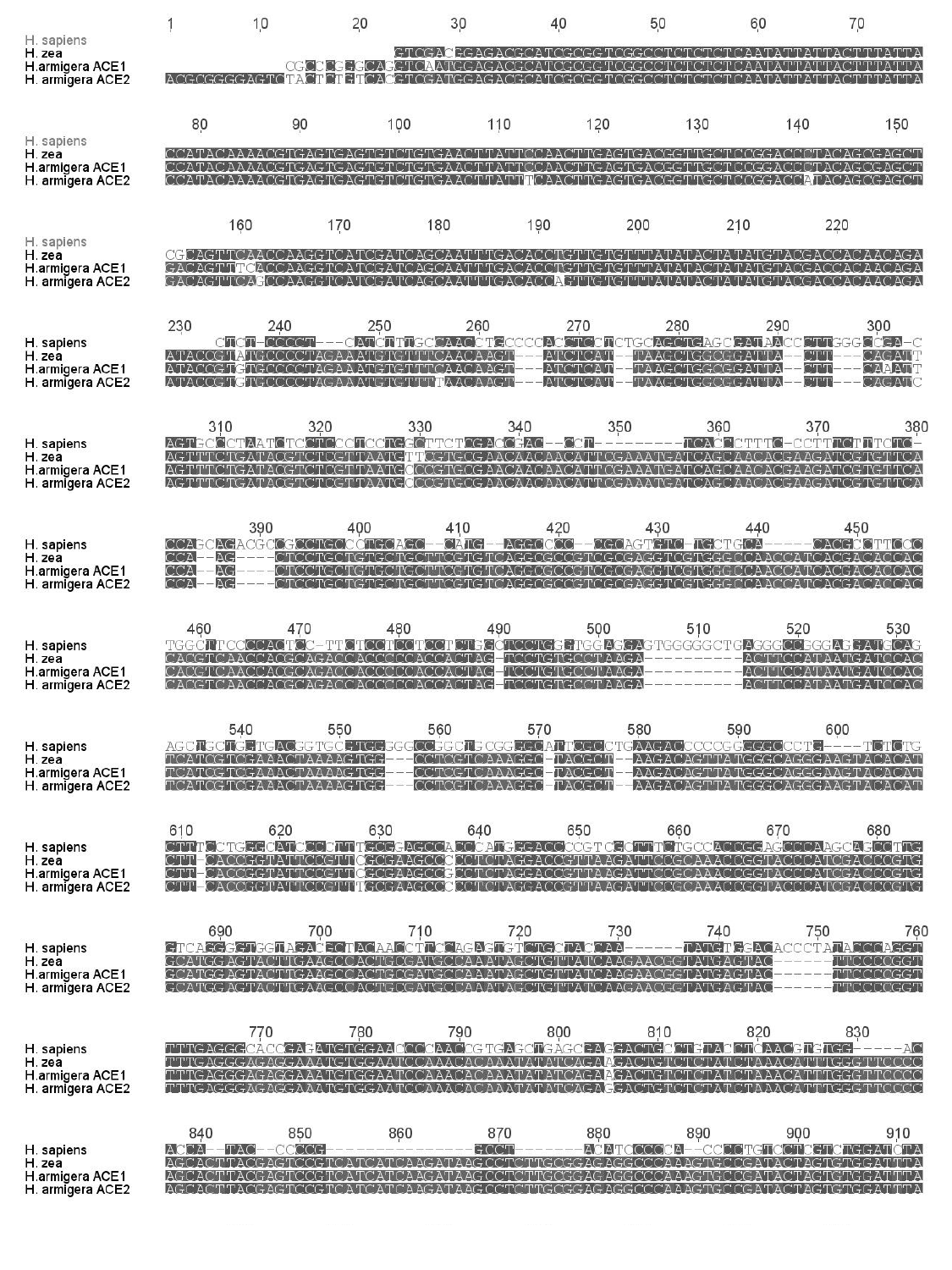

## Slide 2
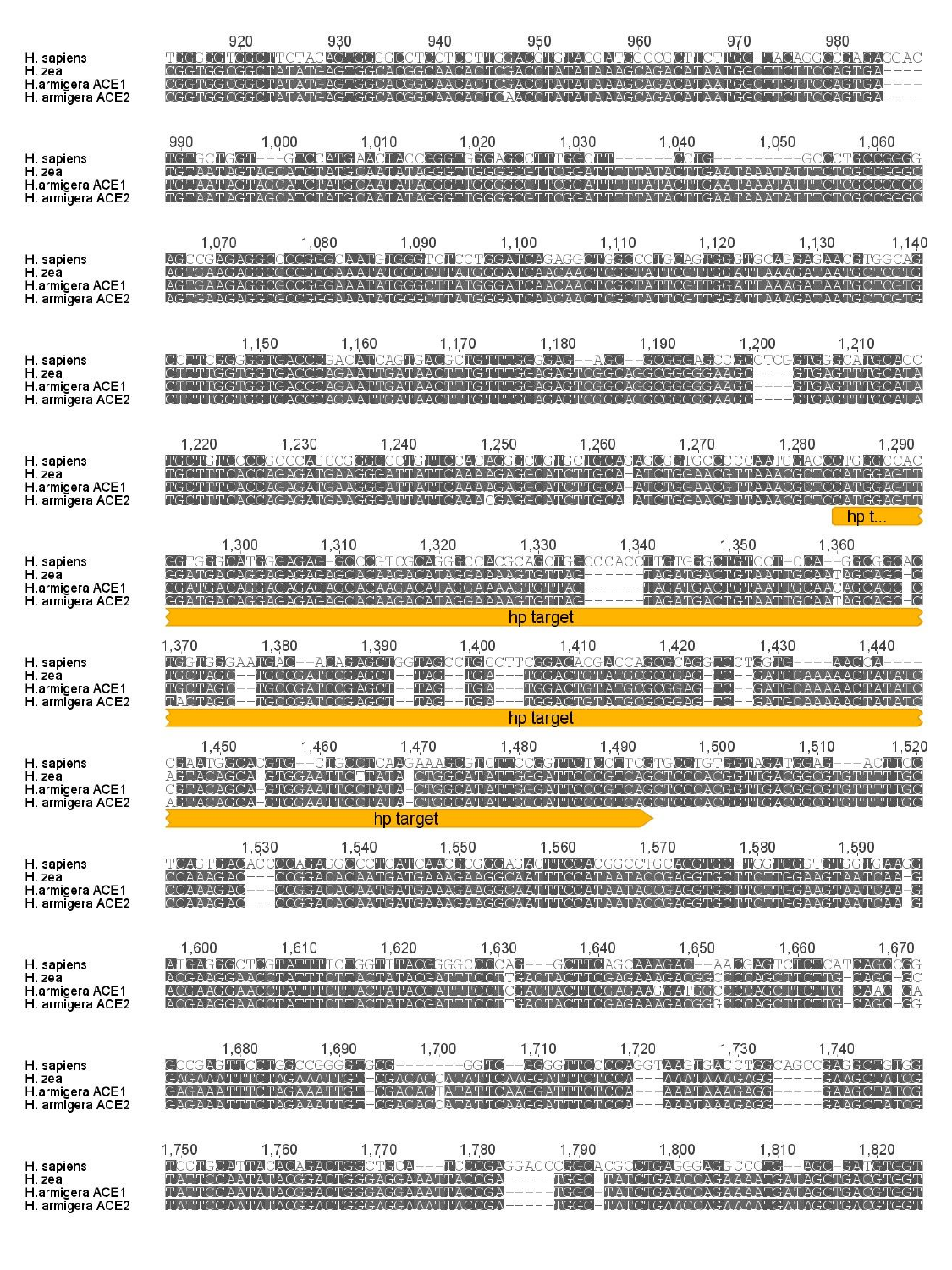

## Slide 3
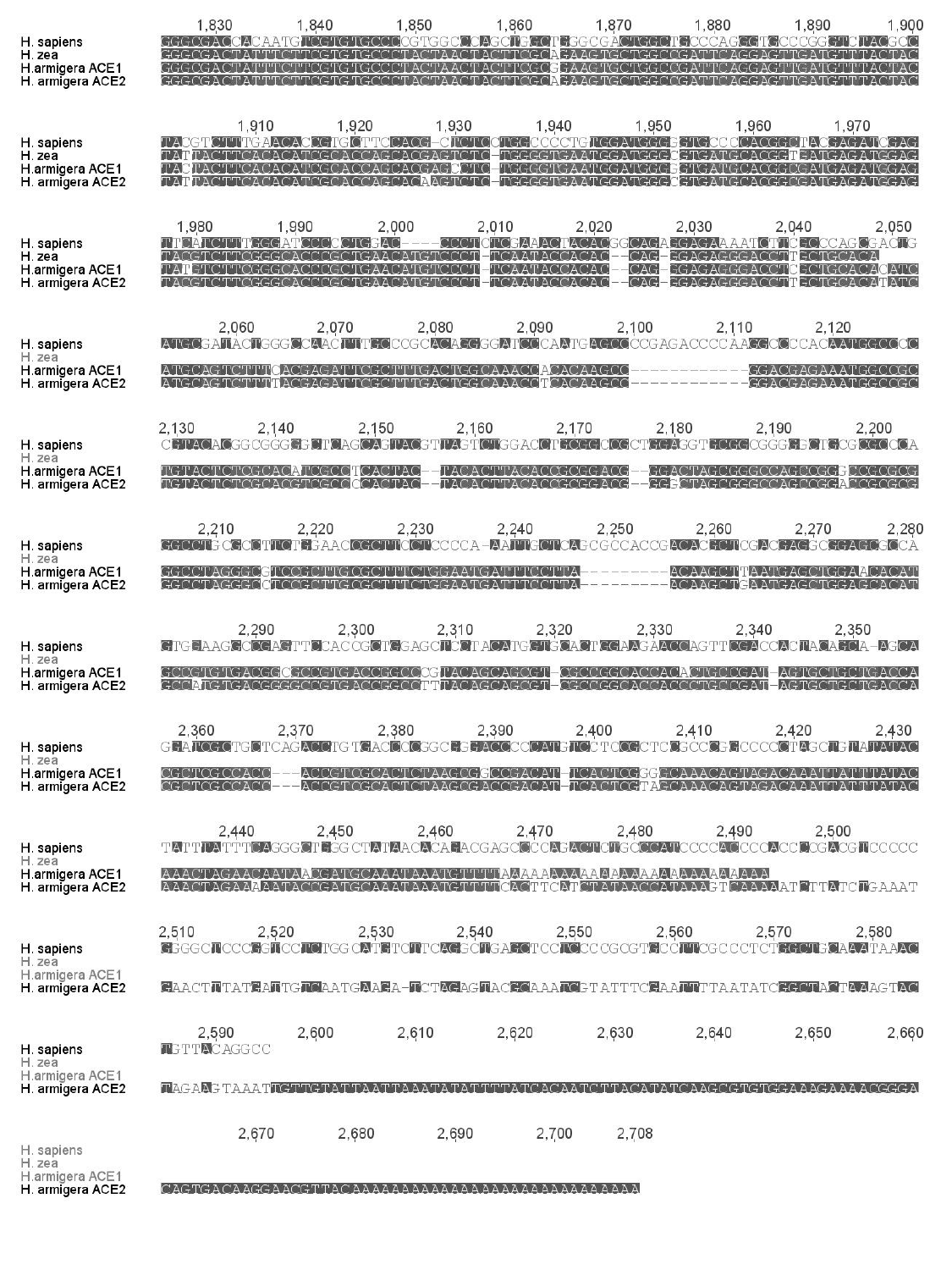

## Slide 4
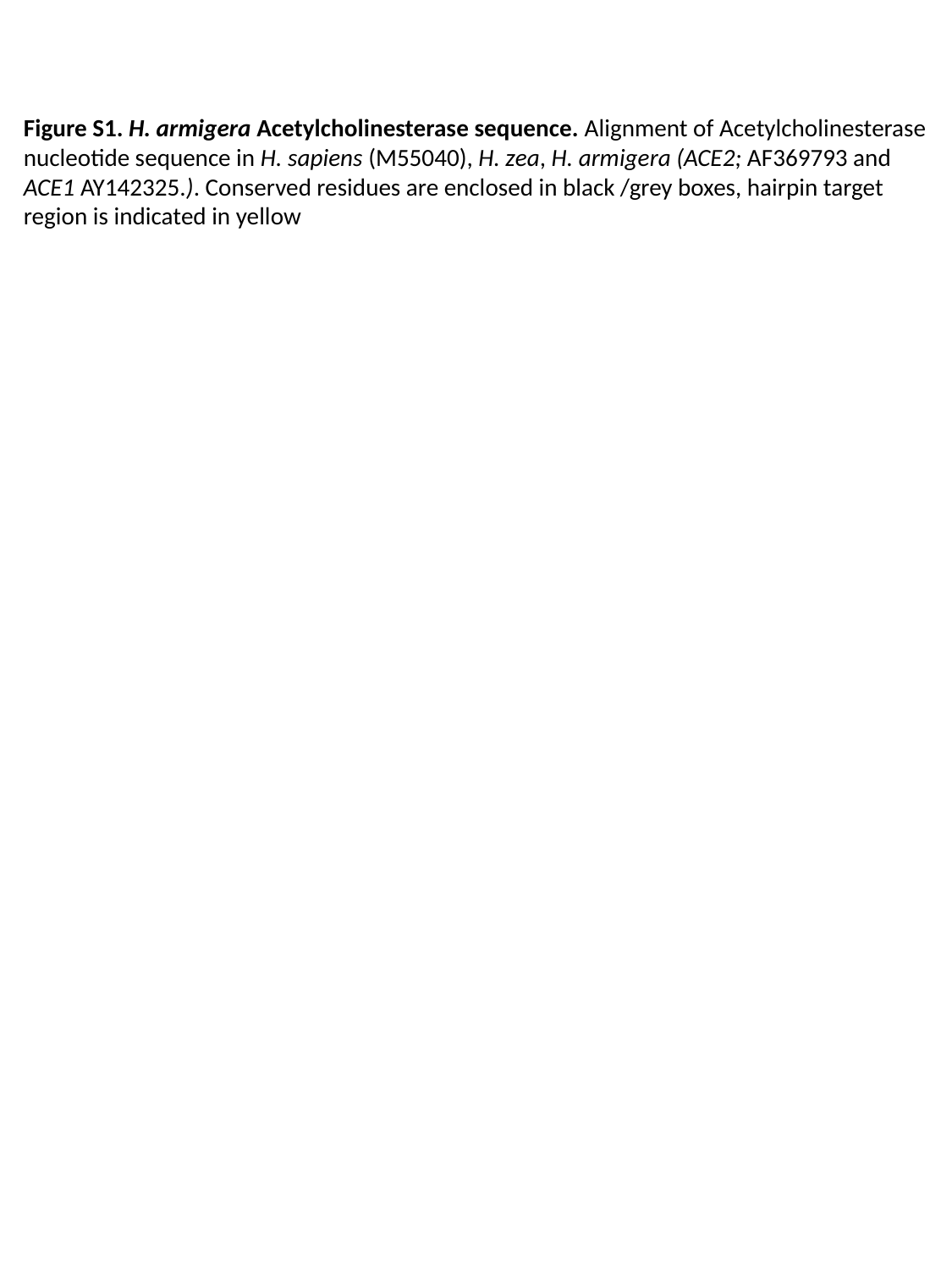

Figure S1. H. armigera Acetylcholinesterase sequence. Alignment of Acetylcholinesterase nucleotide sequence in H. sapiens (M55040), H. zea, H. armigera (ACE2; AF369793 and ACE1 AY142325.). Conserved residues are enclosed in black /grey boxes, hairpin target region is indicated in yellow

## Slide 5
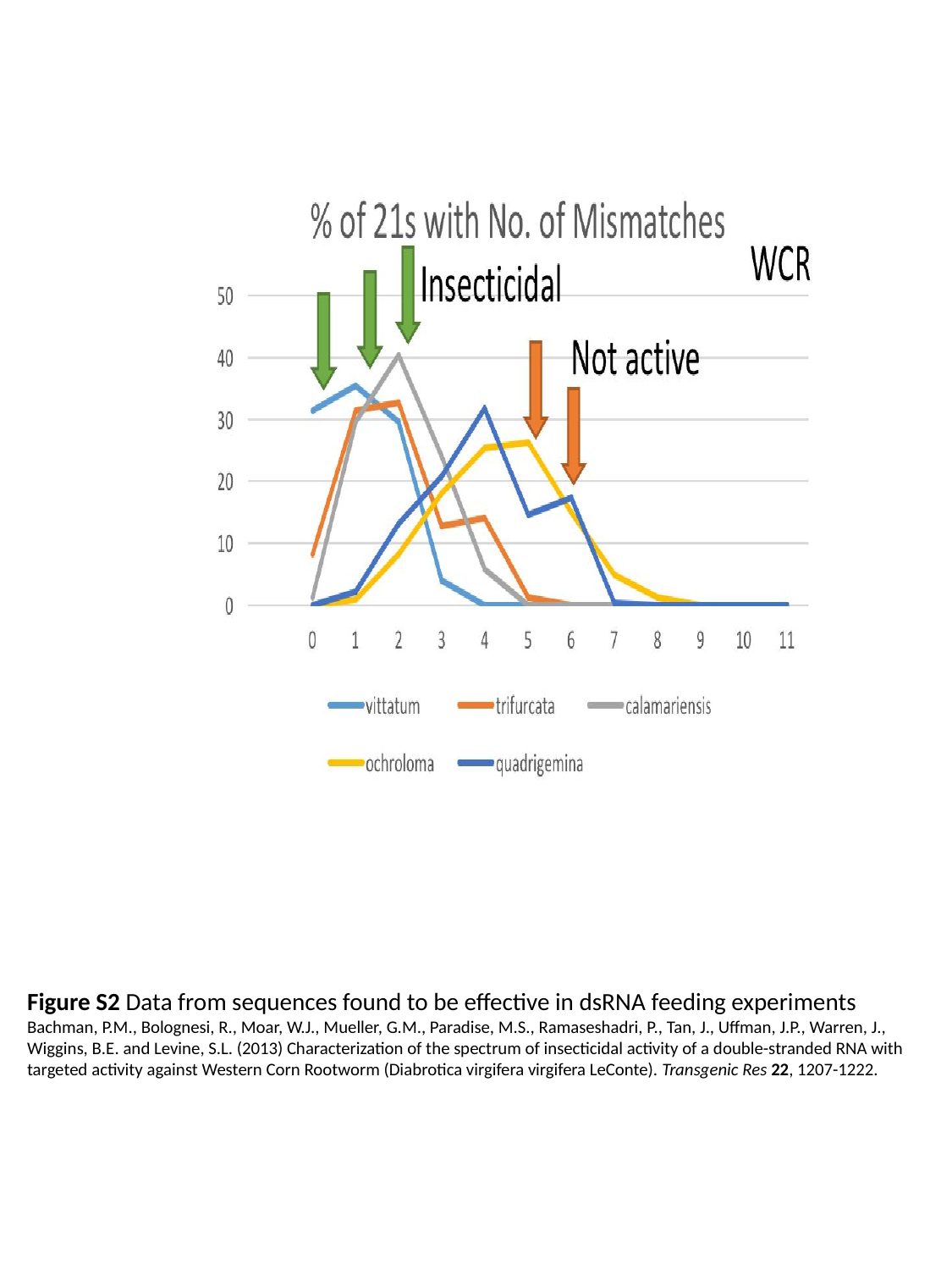

Figure S2 Data from sequences found to be effective in dsRNA feeding experiments
Bachman, P.M., Bolognesi, R., Moar, W.J., Mueller, G.M., Paradise, M.S., Ramaseshadri, P., Tan, J., Uffman, J.P., Warren, J., Wiggins, B.E. and Levine, S.L. (2013) Characterization of the spectrum of insecticidal activity of a double-stranded RNA with targeted activity against Western Corn Rootworm (Diabrotica virgifera virgifera LeConte). Transgenic Res 22, 1207-1222.

## Slide 6
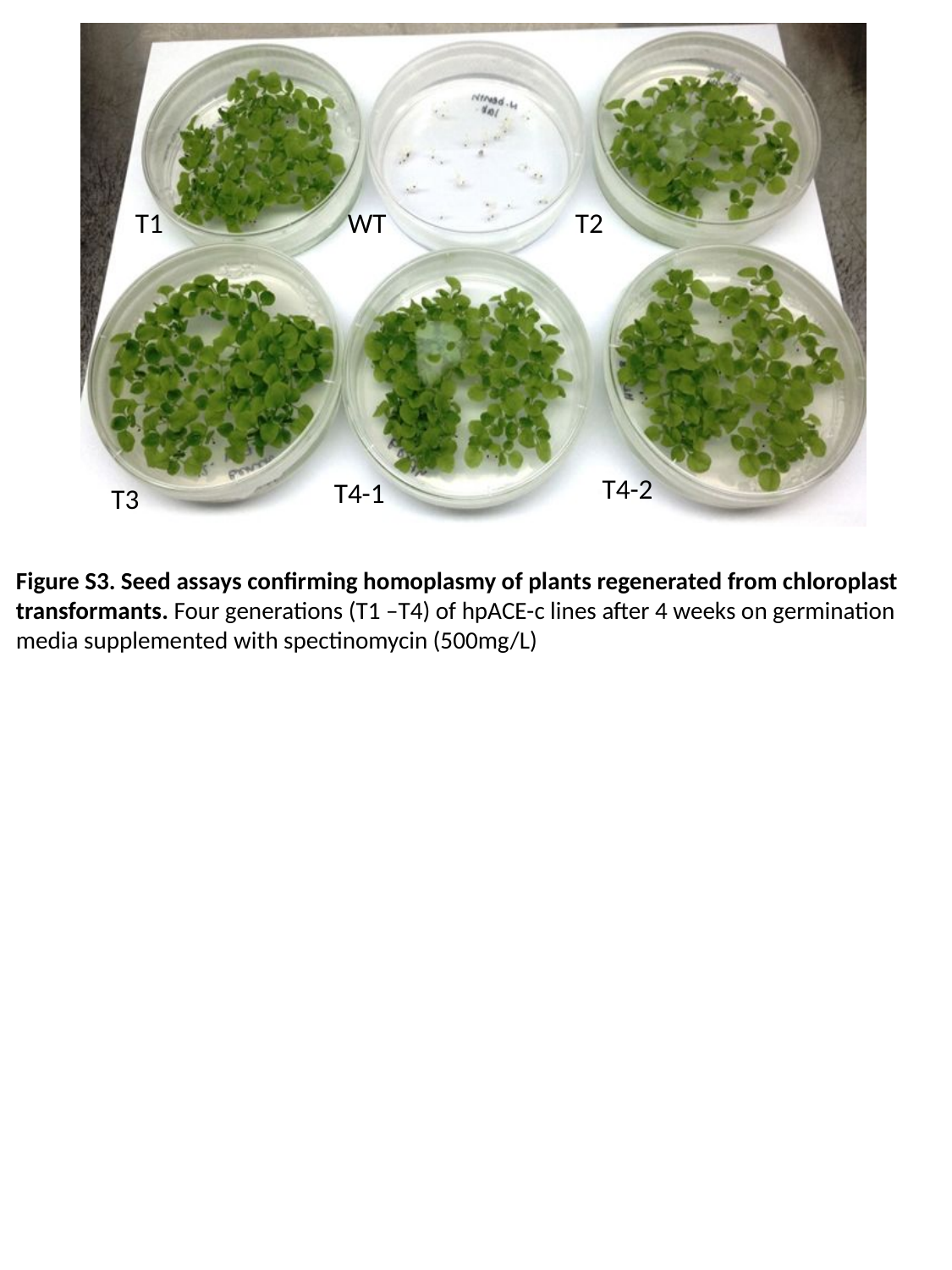

T1
WT
T2
T4-2
T4-1
T3
Figure S3. Seed assays confirming homoplasmy of plants regenerated from chloroplast transformants. Four generations (T1 –T4) of hpACE-c lines after 4 weeks on germination media supplemented with spectinomycin (500mg/L)

## Slide 7
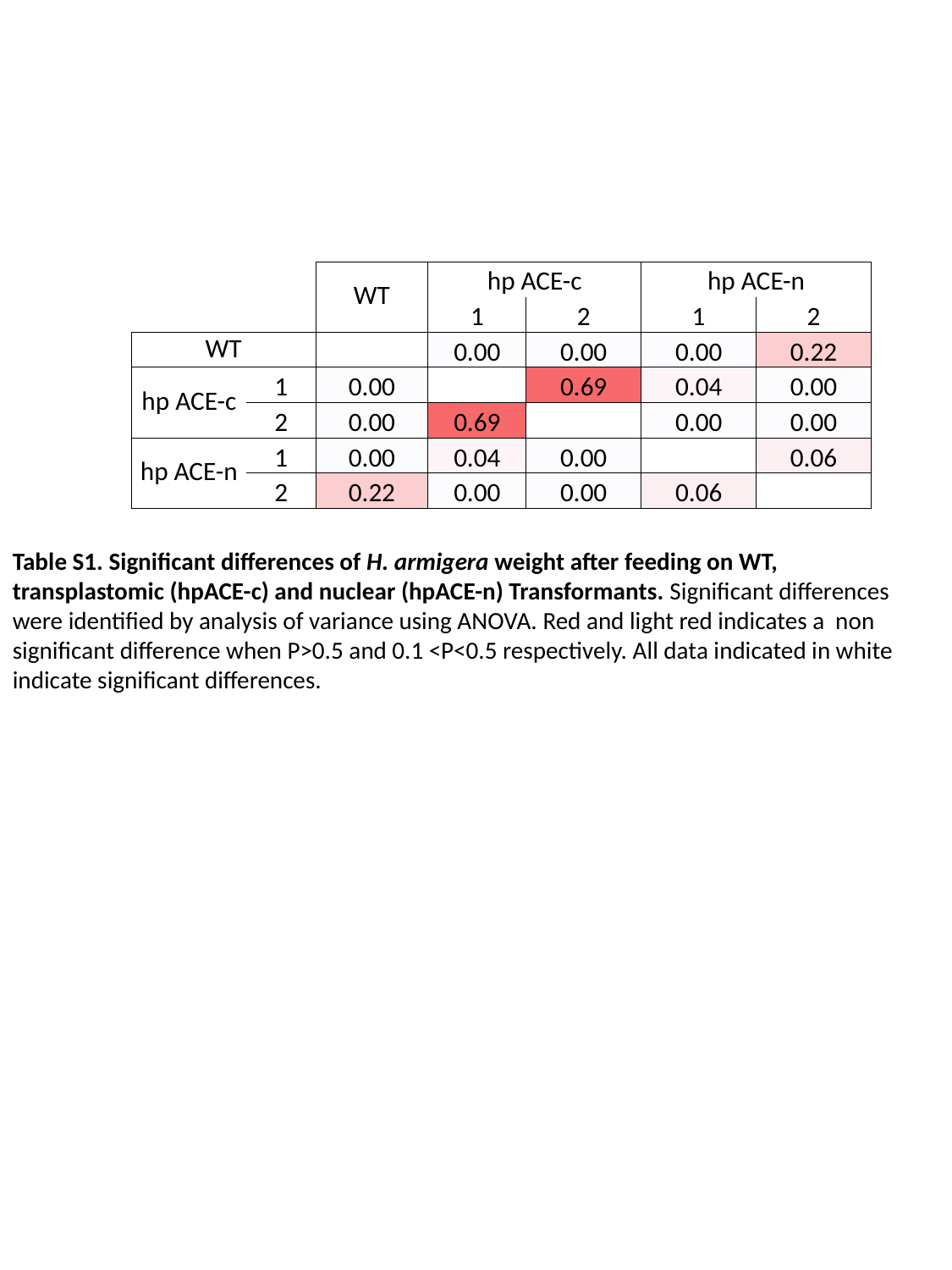

| | | WT | hp ACE-c | | hp ACE-n | |
| --- | --- | --- | --- | --- | --- | --- |
| | | | 1 | 2 | 1 | 2 |
| WT | | | 0.00 | 0.00 | 0.00 | 0.22 |
| hp ACE-c | 1 | 0.00 | | 0.69 | 0.04 | 0.00 |
| | 2 | 0.00 | 0.69 | | 0.00 | 0.00 |
| hp ACE-n | 1 | 0.00 | 0.04 | 0.00 | | 0.06 |
| | 2 | 0.22 | 0.00 | 0.00 | 0.06 | |
Table S1. Significant differences of H. armigera weight after feeding on WT, transplastomic (hpACE-c) and nuclear (hpACE-n) Transformants. Significant differences were identified by analysis of variance using ANOVA. Red and light red indicates a non significant difference when P>0.5 and 0.1 <P<0.5 respectively. All data indicated in white indicate significant differences.

## Slide 8
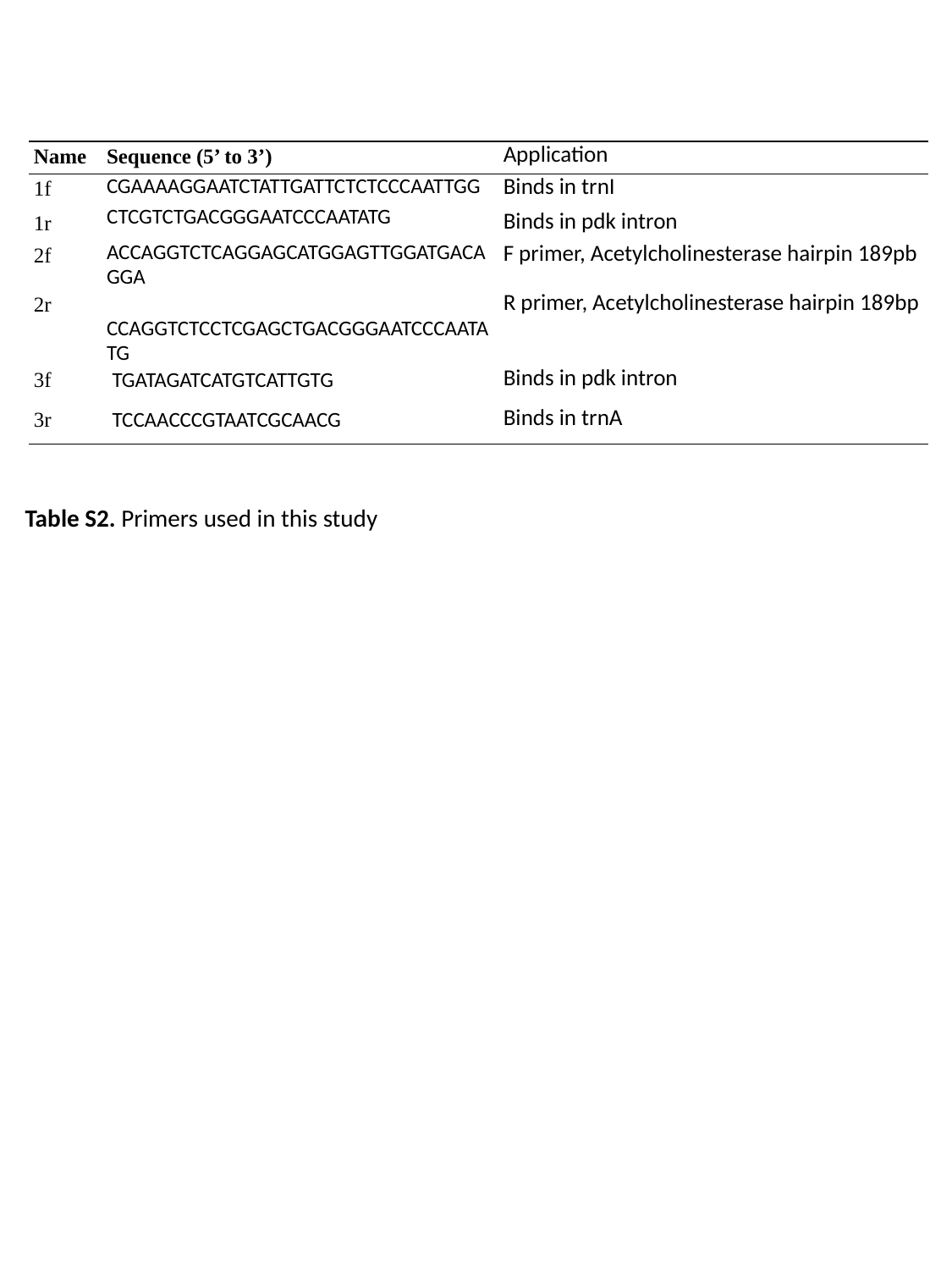

| Name | Sequence (5’ to 3’) | Application |
| --- | --- | --- |
| 1f | CGAAAAGGAATCTATTGATTCTCTCCCAATTGG | Binds in trnI |
| 1r | CTCGTCTGACGGGAATCCCAATATG | Binds in pdk intron |
| 2f | ACCAGGTCTCAGGAGCATGGAGTTGGATGACAGGA | F primer, Acetylcholinesterase hairpin 189pb |
| 2r | CCAGGTCTCCTCGAGCTGACGGGAATCCCAATATG | R primer, Acetylcholinesterase hairpin 189bp |
| 3f | TGATAGATCATGTCATTGTG | Binds in pdk intron |
| 3r | TCCAACCCGTAATCGCAACG | Binds in trnA |
Table S2. Primers used in this study
